# Reconstruction of a Giant Gluteal Neurofibroma Using an Advancement Flap and Gluteal Lift in Neurofibromatosis Type 1

**DOI:** 10.7759/cureus.113991

**Published:** 2026-08-05

**Authors:** Arym P Preza Estrada, Silvia C Prado Rico, Dan Palacios Alvarez, Daniel Chalini Mazariegos, Oscar Luis Verdugo Briseño

**Affiliations:** 1 Plastic and Reconstructive Surgery, Hospital Regional Dr. Valentín Gómez Farías - Instituto de Seguridad y Servicios Sociales de los Trabajadores del Estado (ISSSTE), Guadalajara, MEX; 2 General Surgery, Instituto de Seguridad y Servicios Sociales de los Trabajadores del Estado (ISSSTE), La Paz, MEX; 3 Plastic and Reconstructive Surgery, Instituto de Seguridad y Servicios Sociales de los Trabajadores del Estado (ISSSTE), La Paz, MEX

**Keywords:** advancement flap, cutaneous neurofibromas, gluteal surgery, neurofibromatosis type 1, plastic surgergy

## Abstract

Neurofibromatosis type 1 (NF1) is an autosomal dominant disorder characterized by the development of multiple neurofibromas that may cause pain, functional impairment, and significant aesthetic deformity. Extensive gluteal lesions represent a reconstructive challenge because of poor tissue quality, wound tension, and the high risk of postoperative wound complications. We report the case of a 43-year-old man with NF1 who presented with a giant left gluteal cutaneous neurofibroma causing marked soft-tissue deformity. The lesion was completely excised, and the resulting defect was reconstructed using local advancement flaps combined with gluteal lifting. Although postoperative wound dehiscence occurred, it was successfully managed with conservative treatment, resulting in complete wound healing and satisfactory functional and aesthetic outcomes. This case highlights the importance of individualized reconstructive planning and demonstrates that combining local advancement flaps with gluteal lifting can provide effective soft-tissue coverage and contour restoration following resection of extensive gluteal neurofibromas, contributing to the limited literature on reconstruction of these uncommon defects.

## Introduction

Neurofibromatosis type 1 (NF1) is a rare genetic disorder inherited in an autosomal dominant pattern and characterized by complete penetrance. Its estimated incidence is approximately one per 3,000 live births. Despite its autosomal dominant inheritance, nearly half of all cases arise from de novo pathogenic variants in the NF1 gene. This gene was identified in 1990 and is located on chromosome 17q11.2, where it encodes the tumor suppressor protein neurofibromin [1]. The clinical manifestations of NF1 include multiple café-au-lait macules (CALMs), lentiginous freckling in intertriginous regions that are typically not exposed to sunlight, iris Lisch nodules, nervous system tumors, and a wide range of other associated features. Diagnosis has traditionally been based on the clinical criteria established by the National Institutes of Health (NIH) in 1987 and their subsequent revision in 2021 [2]. Beyond its physical manifestations, NF1 may have a substantial psychosocial impact. Affected individuals may experience anxiety, depression, reduced self-esteem, altered body image, social withdrawal, difficulties in interpersonal relationships, behavioral disturbances, and academic challenges [3].

The surgical management of cutaneous neurofibromas (CNs) in patients with NF1 requires careful planning based on lesion extent, anatomical location, functional impairment, and aesthetic considerations [4,5]. Large sacrogluteal neurofibromas requiring complex reconstruction are uncommon and present unique reconstructive challenges because of the size of the resulting defect, compromised tissue quality, and the mechanical stress imposed on the gluteal region, making the selection of an appropriate reconstructive strategy essential [5]. Therefore, reporting reconstructive approaches for these uncommon defects may help expand the limited surgical literature and provide practical guidance for similar cases. The purpose of this report is to describe the surgical management of an extensive sacrogluteal neurofibroma reconstructed with an advancement flap combined with gluteal lifting, highlighting the reconstructive decision-making process, postoperative wound management, and clinical outcome.

## Case presentation

In October 2024, a 43-year-old man with hereditary NF1 presented with a long-standing progressive enlargement of the left gluteal region that had been present since adolescence and extended into the ipsilateral thigh. He reported marked gluteal asymmetry and excess skin in the affected area but denied urinary or gastrointestinal symptoms. His medical history was notable for chronic tobacco use, discontinued two months before surgery, and class I obesity, with a body weight of 103 kg during follow-up.

**Figure 1:**
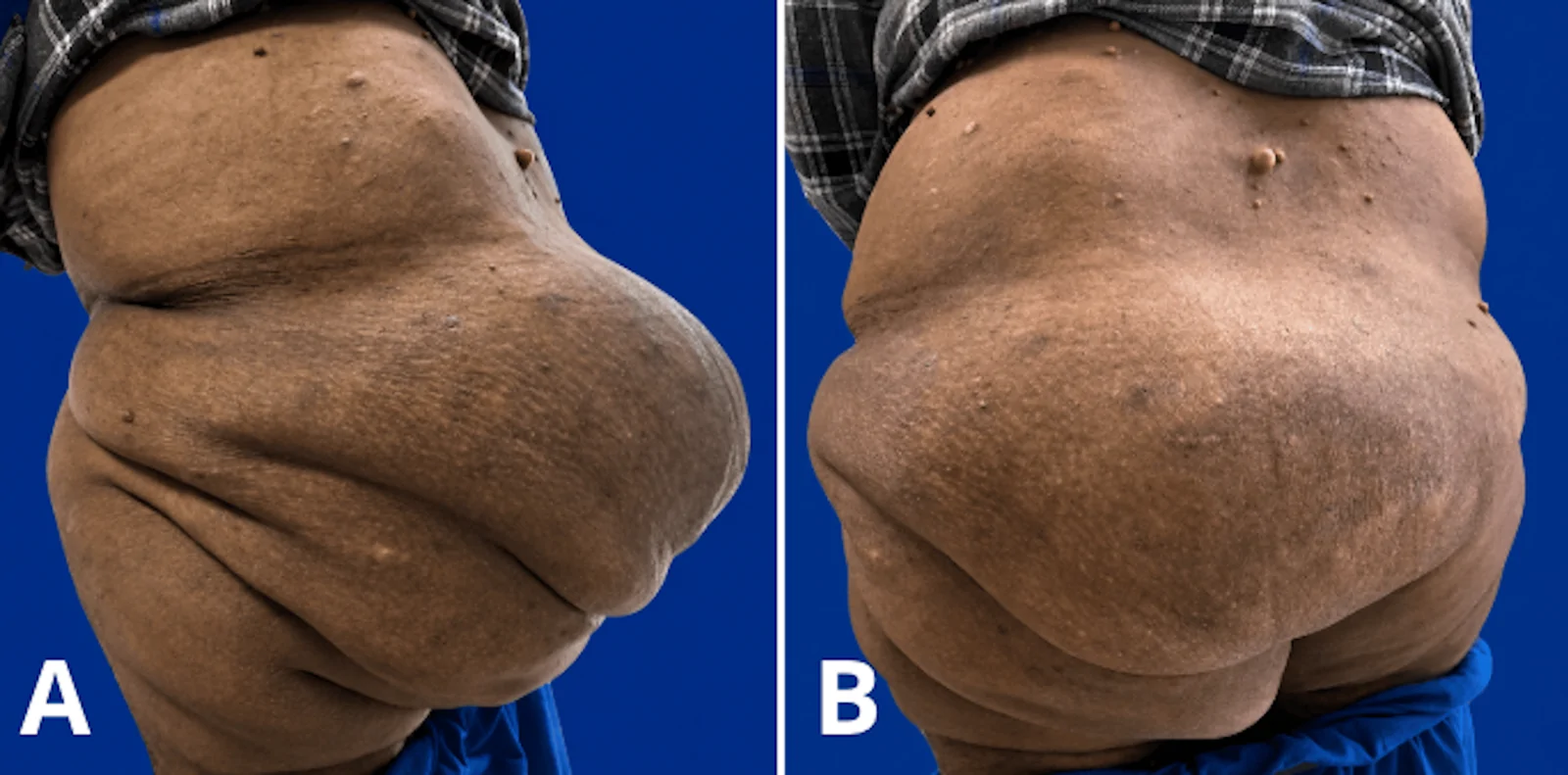
Preoperative clinical appearance of the giant gluteal neurofibroma. (A) Left lateral view demonstrating the giant gluteal neurofibroma. (B) Posterior view showing the marked gluteal asymmetry and contour deformity involving both gluteal regions secondary to the mass.

Physical examination revealed a predominantly left-sided mass involving the sacral and gluteal regions with extension into the ipsilateral thigh, measuring approximately 70 × 50 cm. The lesion was soft, mobile, well-circumscribed, and appeared to originate from the subcutaneous tissue. The overlying skin demonstrated hyperpigmentation, cutaneous laxity, and multiple pedunculated nodular lesions consistent with neurofibromas. Marked gluteal asymmetry with deviation of the intergluteal cleft toward the left side was observed (Figure 1).

Contrast-enhanced computed tomography demonstrated a large heterogeneous soft-tissue mass infiltrating the gluteal musculature and extending into the posterior aspect of the thigh, without evidence of lymphadenopathy or other associated abnormalities. The original CT images were unavailable because they had been removed from the institutional imaging archive in accordance with the hospital data retention policy.

Based on the clinical and radiological findings, surgical treatment was planned. The patient underwent complete resection of the sacrogluteal tumor followed by reconstruction using an advancement flap combined with gluteal lifting. The surgical specimen weighed approximately 6 kg and had a multilobulated appearance. It was located predominantly within the subcutaneous plane, with focal infiltration of fibers of the gluteus maximus muscle. During dissection, the lesion exhibited marked vascularity, with multiple feeding vessels, including arterioles measuring approximately 1.0 to 1.5 cm in diameter. Estimated intraoperative blood loss was 3,000 mL, requiring transfusion of two units of packed red blood cells and three units of fresh frozen plasma, in addition to temporary vasopressor support, which was discontinued before discharge from the post-anesthesia care unit.

**Figure 2:**
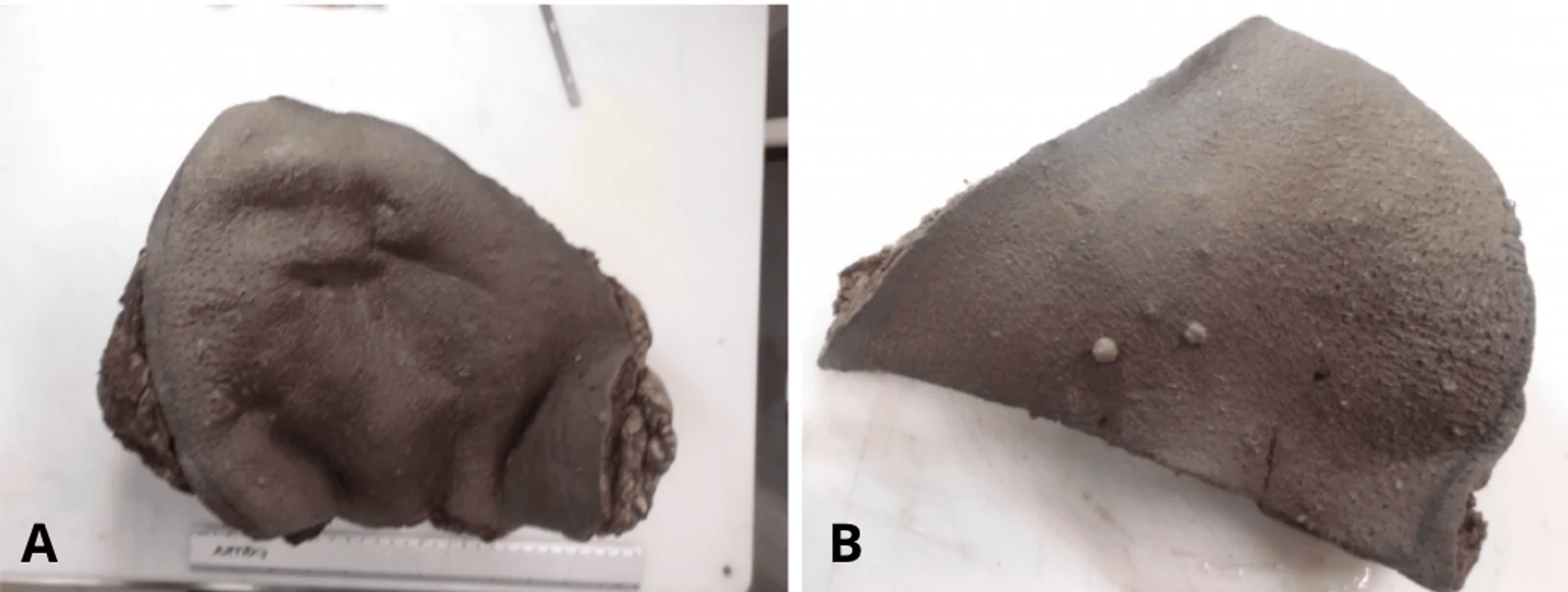
Gross pathological specimen and histopathological findings. (A) First surgical specimen (30 × 25 cm) with intact overlying skin and mild induration. (B) Second surgical specimen (26 × 23 cm) with similar gross appearance. Histopathological examination confirmed a completely excised pigmented cutaneous neurofibroma, characterized by a benign Schwann cell proliferation involving the dermis and subcutaneous tissue, without atypia, mitotic activity, or malignant features.

Histopathological examination confirmed a completely excised pigmented CN of the gluteal region. The gross pathological specimen is shown in Figure 2. Histopathological photomicrographs were unavailable because they had not been archived by the Department of Pathology; however, the diagnosis was confirmed based on the original pathology report.

In November 2024, approximately one month after surgery, the patient developed wound dehiscence at the central confluence of the surgical incision, extending toward the inferior aspect of the intergluteal cleft. Physical examination revealed a wound dehiscence measuring approximately 3 cm in length and 5 cm in depth, with healthy granulation tissue at the wound base, irregular wound edges, and no clinical signs of infection.

**Figure 3:**
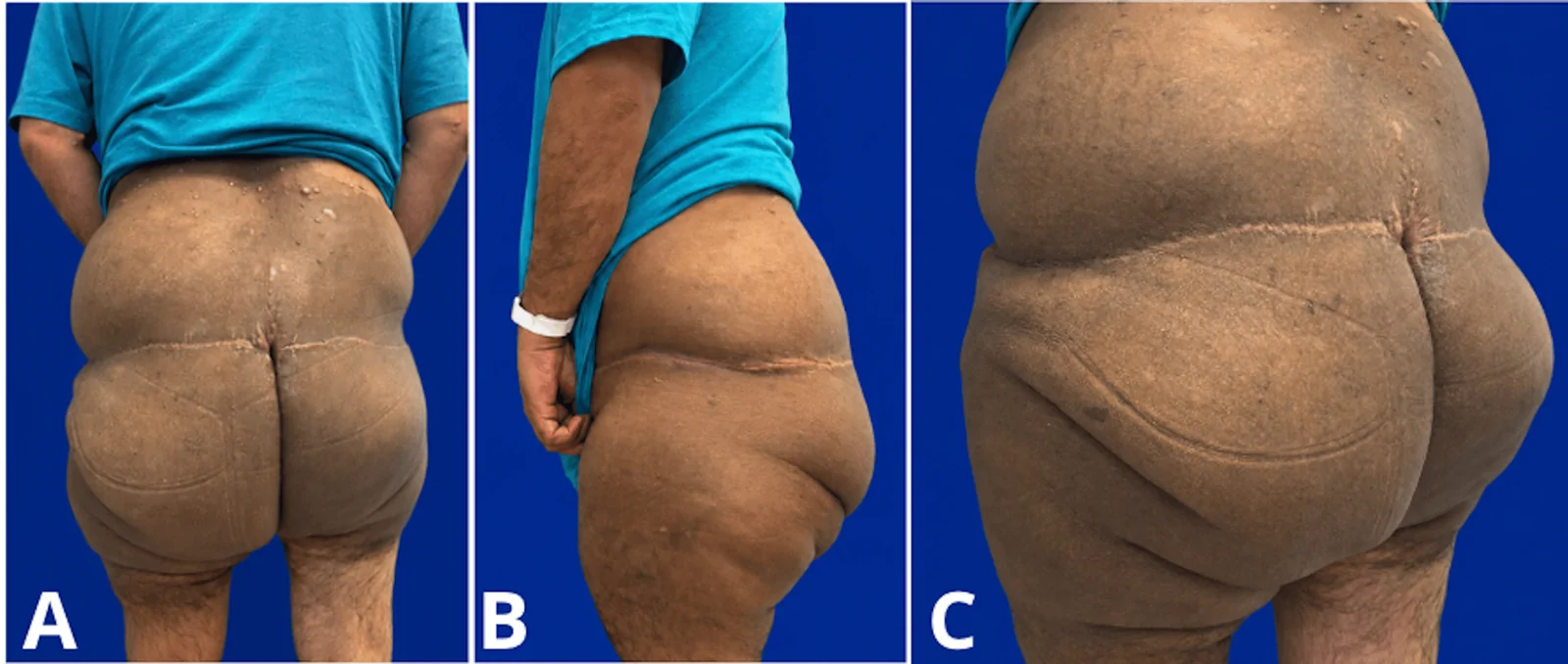
Postoperative outcome following giant gluteal neurofibroma resection and reconstruction with an advancement flap and gluteal lift. (A) Posterior view demonstrating restoration of the left gluteal contour with satisfactory wound healing, without evidence of wound dehiscence. (B) Left lateral view showing the reconstructed gluteal region with adequate soft tissue coverage and favorable contour. (C) Left posterolateral (45°) view illustrating the postoperative symmetry and contour of the reconstructed gluteal region.

**Figure 4:**
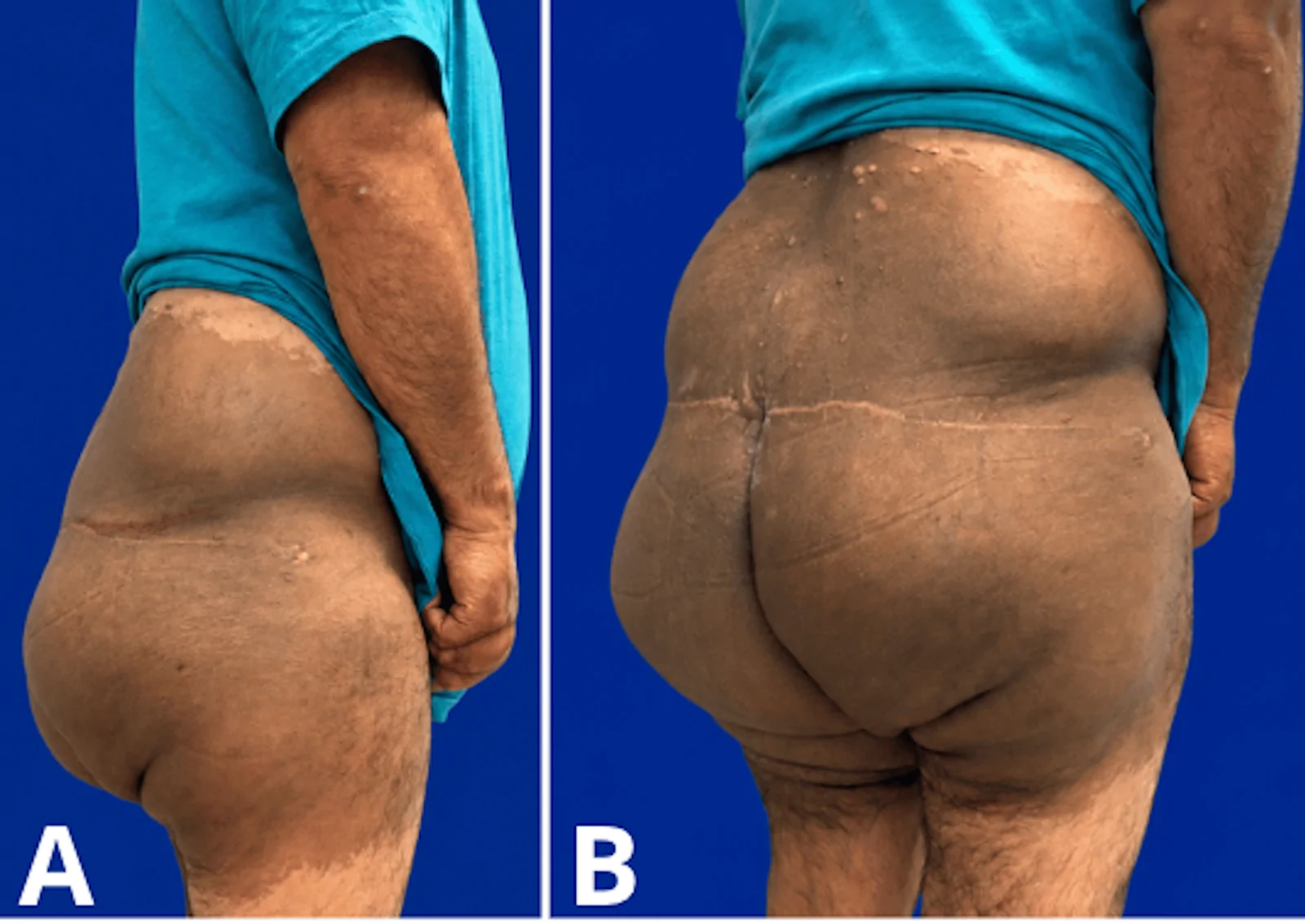
Postoperative appearance of the right gluteal region following reconstruction. (A) Right lateral view demonstrating satisfactory wound healing with a well-healed surgical scar and no evidence of wound dehiscence. (B) Right posterolateral (45°) view showing preservation of the gluteal contour and favorable postoperative aesthetic outcome.

Conservative management was initiated with oral protein supplementation, topical tranexamic acid powder, serial local wound care, and calcium alginate dressings. After an adequate wound bed had developed, tertiary wound closure was performed. However, partial wound dehiscence recurred during follow-up and was managed successfully with continued local wound care. The postoperative course was ultimately favorable, with progressive granulation tissue formation, gradual wound contraction, and complete epithelialization, resulting in complete wound healing. At the six-month follow-up, there was no evidence of active infection or tumor recurrence. The patient reported improved comfort while sitting and walking, easier performance of daily activities and personal hygiene, as well as satisfaction with the postoperative contour of the gluteal region (Figures 3-4).

## Discussion

NF1 was first recognized as a systemic disorder following its description by Friedrich von Recklinghausen in 1882 [4]. The understanding and classification of the disease subsequently evolved, particularly after the 1987 NIH Consensus Development Conference, which established a standardized framework for its nomenclature and clinical diagnosis [2]. Beyond its characteristic cutaneous manifestations, NF1 is associated with an increased susceptibility to several malignancies, including malignant peripheral nerve sheath tumors (MPNSTs), central nervous system tumors, and breast cancer in women younger than 50 years. This broad clinical spectrum emphasizes the systemic nature of NF1 and the need for comprehensive evaluation beyond its cutaneous manifestations [4].

CNs are benign dermal tumors resulting from biallelic loss of the NF1 gene in Schwann cell-like tumor cells and are present in approximately 99% of patients with NF1 [5]. Individuals with NF1 frequently identify these skin tumors as one of the principal factors negatively affecting their mental health. Studies evaluating the psychological impact of NF1 in children and adolescents have demonstrated impaired self-image, discrepancies between caregiver and patient perceptions of quality of life, and increased levels of anxiety compared with healthy individuals. Similar findings have also been reported in adults. The physical deformity associated with these lesions may lead to embarrassment, concealment of the skin with oversized clothing, difficulties with intimacy, and socioeconomic disadvantages related to reduced opportunities for client-facing occupations. Consequently, the impact of these lesions extends beyond the physical manifestations of the disease and significantly affects psychosocial well-being and quality of life [3,5].

NF1 is caused by loss-of-function variants in the NF1 gene, resulting in reduced levels of functional neurofibromin throughout the body. Neurofibromin is a widely expressed tumor suppressor protein that negatively regulates Ras-mediated signaling. Loss of neurofibromin function leads to dysregulation of these pathways, promoting abnormal cellular proliferation and contributing to the development of the benign and malignant neoplasms associated with NF1 [6].

In addition to their psychosocial impact, CNs may produce clinically significant symptoms. Although these tumors are benign, their raised appearance may produce pruritus, occasionally associated with cutaneous mastocytosis, as well as pain caused by friction against clothing or pressure during movement. For many patients, these symptoms can interfere with daily activities and social interactions [6].

CNs are composed predominantly of Schwann cells, along with fibroblasts, mast cells, pericytes, and perineural cells. Their heterogeneous cellular composition and variable clinical presentation may contribute to the complexity of treatment planning [4].

CNs exhibit a rich vascular network composed of small arterioles and arterial and venous capillaries. These vessels are lined by a single endothelial layer and may contain subendothelial smooth muscle cells or pericytes, with surrounding vascular basement membranes and thickened adventitial tissue [4]. This prominent vascularity represents an important surgical consideration during the excision of extensive lesions, as it may contribute to considerable intraoperative blood loss, consistent with the findings observed in the present case.

The management of CNs remains challenging for both patients and healthcare providers. Recurrence may occur following incomplete resection or even at sites of suture penetration, potentially due to the activation of injury-responsive pluripotent Schwann cell precursors. Surgical excision remains the primary treatment modality for CNs. Although excision is effective for isolated lesions, the high number and variable size of CNs often make complete surgical management impractical. As a result, treatment strategies frequently focus on symptomatic lesions or those with significant aesthetic or functional impact [2,4].

In cases involving extensive gluteal tumors, reconstruction may require not only restoration of soft-tissue coverage but also correction of contour deformities and asymmetry caused by the lesion itself. Gluteal lifting techniques have been widely used to improve gluteal contour through tissue mobilization, correction of tissue redundancy, and redefinition of the gluteal sulcus. Similarly, autologous flap-based gluteal remodeling has demonstrated favorable aesthetic outcomes by restoring projection, symmetry, and overall contour while preserving regional anatomy [7,8]. In the present case, reconstruction with an advancement flap combined with gluteal lifting contributed to correcting the marked asymmetry caused by the tumor and improving the postoperative contour.

Resection of extensive neurofibromatous lesions may result in substantial cutaneous and soft-tissue defects requiring reconstructive procedures. Flap-based reconstruction provides well-vascularized tissue for defect coverage while contributing to the preservation of function and optimization of aesthetic outcomes [9,10]. Therefore, reconstructive planning is particularly important when lesions involve anatomically and aesthetically demanding regions.

The anatomy of the cutaneous vasculature plays a critical role in flap viability and successful reconstruction [9]. Advancement flaps represent a useful reconstructive option because they recruit adjacent well-vascularized tissue, redistribute tension, and preserve tissue characteristics similar to those of the recipient site [10]. These characteristics supported their use in the present case, in which extensive resection required durable coverage while maintaining the contour and native qualities of the surrounding gluteal tissue.

Almeida et al. reported favorable outcomes with individualized reconstructive approaches in patients with NF1, emphasizing the importance of tailoring surgical management according to tumor size, location, and tissue involvement. Similar to our case, extensive tumor resection required individualized reconstructive planning to restore contour and function while minimizing morbidity [5].

The present case demonstrates that the management of extensive gluteal neurofibromas requires an individualized reconstructive strategy that addresses not only soft-tissue coverage but also the functional and aesthetic deformities resulting from the lesion and its resection. Although postoperative wound dehiscence occurred, continued wound management ultimately resulted in complete healing. These findings emphasize the importance of careful preoperative planning and individualized technique selection according to lesion extent, anatomical location, tissue characteristics, and anticipated postoperative morbidity.

## Conclusions

Neurofibroma resection in patients with NF1 represents a reconstructive challenge, particularly in the sacral and gluteal regions, where local moisture, mechanical stress, and compromised tissue quality may increase the risk of wound complications such as dehiscence. This case highlights the importance of individualized surgical planning and suggests that advancement flap reconstruction combined with gluteal lifting may provide adequate soft tissue coverage and contour restoration following resection of extensive neurofibromatous lesions in selected patients. Although this experience is limited to a single case, it illustrates the value of applying reconstructive principles and individualized postoperative management to optimize outcomes in these complex defects.
